# Constructing a novel family of halogen-doped covalent triazine-based frameworks as efficient metal-free photocatalysts for hydrogen production†

**DOI:** 10.1039/c9na00089e

**Published:** 2019-05-18

**Authors:** Zhi Cheng, Kaiyun Zheng, Guiyun Lin, Shengqiong Fang, Liuyi Li, Jinhong Bi, Jinni Shen, Ling Wu

**Affiliations:** Department of Environmental Science and Engineering, Fuzhou University Fuzhou 350108 China bijinhong@fzu.edu.cn; State Key Laboratory of Photocatalysis on Energy and Environment, Fuzhou University Fuzhou 350108 China; Key Laboratory of Eco-materials Advanced Technology, Fuzhou University Fuzhou 350108 China lyli@fzu.edu.cn

## Abstract

Halogens, as typical non-metal dopants, have attracted intensive interests for developing highly active photocatalysts. However, the essential factors and underlying mechanism of halogen modification are still unclear. Herein, we systematically report the development of halogen (F, Cl and Br)-doped covalent triazine-based frameworks (CTFs) *via* a facile thermal treatment of CTFs and an excess of ammonium halide. The introduction of halogen atoms endowed CTFs with multiple superior effects such as improved optical absorption, promoted charge migration, narrowed band gaps and tuned band positions. The newly developed halogen-doped CTFs showed remarkable photocatalytic activities for H_2_ evolution under visible-light irradiation. Notably, the most enhanced photocatalytic performance was obtained with Cl-doped CTFs, which exhibited 7.1- and 2.4-fold enhancements compared to un-doped CTFs and Cl-doped g-C_3_N_4_, respectively. The electronegativity and atomic radius of the halogen atoms affected the modification of the optical and electronic properties, leading to different photocatalytic performances of F-, Cl- and Br-doped CTFs. The conclusions presented in this work will provide some new insights into the understanding of the doping effect for the improvement of the photocatalytic activity of halogen-doped CTF photocatalysts.

## Abstract 2

Developing metal-free halogen-doped covalent triazine-based frameworks for enhanced visible-light-driven photocatalytic H_2_-production performances.

## Graphical abstract

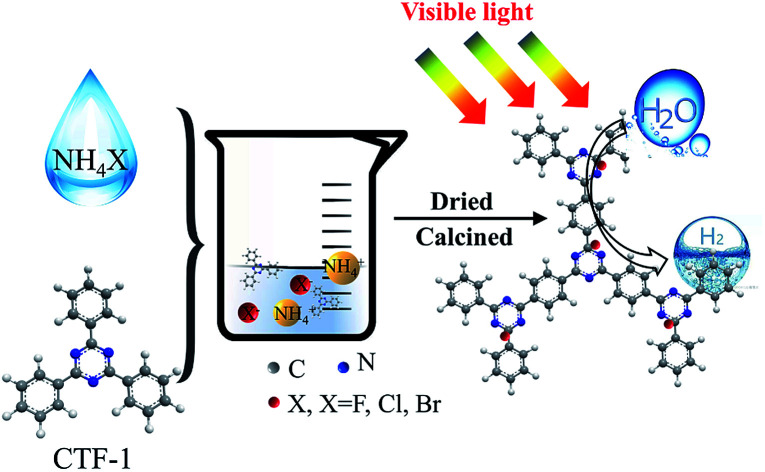

## Introduction

Conjugated polymer semiconductors have been investigated as promising photocatalysts due to their diverse molecular structures, high surface areas and adjustable electronic band structures.^1–3^ Moreover, conjugated polymers are mainly composed of abundant carbon, nitrogen and oxygen elements and are more economical than conventional inorganic semiconductors. Recently, a few conjugated polymer semiconductors have been explored as visible-light-driven photocatalysts for hydrogen generation.^4–7^ Covalent triazine-based frameworks (CTFs), as a type of nitrogen-rich conjugated polymer sharing similar triazine building blocks with carbon nitride, have attracted intense attention.^8–11^ The π-conjugated structure of CTFs is favourable for visible-light absorption, and the π-stacked aromatic units are expected to promote the separation and transfer of photoinduced charge carriers, which are beneficial for the photocatalytic reaction.^12^ Zhao and coworkers investigated the electronic structures, optical properties and band edge alignment of 2D covalent triazine frameworks (CTFs) using first principles calculations, forecasting that 2D-CTF is a kind of novel visible-light-driven photocatalyst for water splitting.^13^ Their theoretical results stimulated tremendous interest to explore novel CTF-based materials as visible-light-driven photocatalysts. In our previous report, a kind of CTF material (CTF-1) with triazine rings (C_3_N_3_H_3_) as the building units was developed. It exhibited photocatalytic H_2_-production activity under visible-light irradiation;^14^ however, its photocatalytic performance was still unsatisfactory, which was generally due to the limited visible-light absorption and weak charge separation/transfer.

In the field of photocatalysis, the band-gap engineering of conjugated polymers *via* chemical doping plays an important role in modulating the light absorption and electronic structure for the improvement in photocatalytic activity.^15–18^ Non-metal doping can maintain the metal-free property of the conjugated polymers and avoid the thermal variation in the chemical states of the doped metal ions.^19^ Recently, halogen molecules have gained interest as non-metal dopants for enhancing the photocatalytic activity. Although a variety of F-, Cl- and Br-doped conjugated polymer materials with enhanced photocatalytic activities have been investigated,^20–22^ there are still only a few related theoretical and experimental studies that have probed the detailed mechanism and essential factors underlying these halogen doping effects.^23–25^ For instance, Yu *et al.* evaluated the effects of halogen doping on the band structure and electronic and optical properties of the state-of-the-art g-C_3_N_4_ by density functional theory (DFT) calculations. They demonstrated that the halogen-doped g-C_3_N_4_ systems have a narrowed band gap, increased light absorption and a reduced work function, which are conducive to high photocatalytic activity.^26^ Jing *et al.* prepared halogen-modified g-C_3_N_4_ nanosheets to improve the photocatalytic activity for degrading organic pollutants and converting CO_2_ to CH_4_.^25^ Until lately, some key issues such as the origins of different doping effects among the halogen dopants in the frameworks are still unclear. Moreover, the experimental investigation of halogen-doped CTFs has never been reported. As a consequence, a systematic investigation of the doping effect in terms of the electronic structure, optical properties and charge carrier separation/transfer in halogen-doped CTFs is urgently required for a better understanding of their enhancement in photocatalytic activity.

In this work, for the first time, we systematically investigated the electronic structure, optical properties and charge carrier mobility as well as the photocatalytic performance of halogen-doped CTF photocatalysts. A series of halogen-doped (F, Cl and Br) CTFs were synthesized *via* heating the mixture of as-prepared CTFs with excess ammonium halide. The incorporation of halogen atoms exhibited multiform favourable effects, including significantly improved visible-light absorption, accelerated charge carrier separation/transfer, narrowed band gaps and better aligned band structures. Benefiting from the above advantages, the halogen-doped CTFs displayed improved photocatalytic activity for water splitting under visible-light irradiation. It is fascinating to note that the Cl-doped CTFs exhibited the most enhanced photocatalytic performance, which could be ascribed to the suitable electronegativity and atomic radius of the Cl atom among the halogen atoms. This work will provide new insights into the underlying essential factors and detailed mechanisms regarding the enhancement of the photocatalytic activity for halogen-doped CTFs; moreover, it can inspire the design and synthesis of highly efficient CTF-based photocatalysts.

## Experimental section

### Synthesis of CTF-1

CTF-1 was synthesized following the procedure in a previous report.^14^ Trifluoromethanesulfonic acid (10 mL) was slowly injected into terephthalonitrile (1.28 g) in a round-bottom flask at 0 °C under stirring. The viscous solution was stirred at 30 °C for overnight until the solid was formed. After being kept in a static condition for 3 days, the resultant solid was washed with dichloromethane and ammonium hydroxide. The collected product was dispersed into ammonium hydroxide and stirred overnight. After being centrifuged with distilled water and methanol, the resultant solid was refluxed with methanol and dichloromethane and then dried in vacuum at 80 °C for 12 h. Finally, the light yellow powder CTF-1 was obtained.

### Synthesis of halogen-doped CTF-1

Halogen-doped CTF-1 was synthesized *via* a facile thermal treatment of the mixture of the as-prepared CTF-1 and commercially acquired ammonium halide. In a typical procedure, CTF-1 (0.15 g) and ammonium halide (NH_4_F, NH_4_Cl and NH_4_Br) with a mass ratio of 1 : 20 were well dispersed in 30 mL deionized water and sonicated for 30 min. The mixtures were heated at 90 °C to remove water. After calcination at 250 °C for 2 h, the resultant products were refluxed using methanol for 12 h and centrifuged with deionized water to remove residual ammonium halide. The obtained products were dried at 60 °C, collected and denoted as CTFF, CTFCl and CTFBr (collectively designated as CTFX, X = F, Cl and Br). The physical mixture samples of CTF-1 and ammonium halide were prepared and denoted as CTFX-m (X = F, Cl and Br).

### Synthesis of chlorine-doped g-C_3_N_4_

g-C_3_N_4_ was synthesized following a previous report.^27^ In a typical synthesis process, 10 g of melamine was calcined at 550 °C for 4 h at a heating rate of 2.3 °C min^−1^. The obtained solid was denoted as g-C_3_N_4_. The chlorine-doped g-C_3_N_4_ sample was synthesized by the same procedure as that used for CTFX except that CTF-1 was replaced by g-C_3_N_4_. The as-obtained sample was denoted as CNCl.

### Characterization

The morphologies of the as-prepared samples were investigated by transmission electron microscopy (TEM, TECNAI G2 F20) and scanning electron microscopy (SEM, FEI Nova NANO-SEM 230 spectrophotometer). The Brunauer–Emmett–Teller (BET) surface areas were measured with an ASAP 2020 apparatus (Micromeritics Instrument Corp.). Powder X-ray diffractometer (PXRD) measurements were performed on a Rigaku MiniFlex 600 X-ray diffractometer with Ni-filtered Cu-Kα irradiation (*α* = 1.5406 Å). Fourier transform infrared (FT-IR) spectra were recorded on a Thermo Scientific Nicolet iS10 spectrometer using KBr pellets at a resolution of 4 cm^−1^. Solid-state nuclear magnetic resonance (NMR) experiments were carried out on Bruker Avance III 500. X-ray photoelectron spectroscopy (XPS) data were obtained on a PHI Quantum 2000 XPS system equipped with a monochromatic Al Kα X-ray source. All the binding energies were referenced to the C 1s peak (284.6 eV) of the surface adventitious carbon. Solid-state UV-vis diffuse reflectance spectra (UV-vis DRS) were recorded by an Agilent Cary 5000 UV-vis-NIR spectrophotometer. Electron paramagnetic resonance (EPR) spectra were acquired by a Bruker model A300 X-band spectrometer equipped with a Mercury-xenon lamp (LC8, HAMAMATSU PHOTONICS K.K, Japan).

### Photoelectrochemical measurements

To fabricate the working electrode, the well ground sample (5 mg) and *N*,*N*-dimethylformamide (0.5 mL) were mixed under sonication for 4 h. The obtained suspension (10 μL) was dropped onto a piece of fluoride-tin oxide (FTO) glass substrate with a cover area of 0.25 cm^2^, and the uncovered parts of the FTO glass were coated with epoxy. Then, the working electrode was dried at an ambient temperature. The photocurrent was recorded with a CHI650E electrochemical workstation equipped with a conventional three-electrode cell (Chen Hua Instruments, Shanghai, China). A platinum plate electrode and an Ag/AgCl electrode were used as the counter electrode and the reference electrode, respectively. The electrodes were immersed in a 0.2 M Na_2_SO_4_ aqueous solution and illuminated by a 300 W Xe lamp with a 420 nm cut-off filter from the backside. The Mott–Schottky plots and electrochemical impedance spectroscopy (EIS) plots were obtained with a ZAHNER IM6 electrochemical workstation. The Mott–Schottky analysis was carried out in a 0.2 M Na_2_SO_4_ aqueous solution and the EIS analysis was carried out in a 5 mM K_3_[Fe(CN)_6_]/5 mM K_4_[Fe(CN)_6_]/0.1 M KCl mixed aqueous solution. In addition, each measurement was repeated three times under the same conditions.

### Photocatalytic activity evaluation

The photocatalytic activities of the as-prepared samples were evaluated by water splitting under visible-light irradiation in a glass-enclosed gas-circulation system and a 100 mL Pyrex glass reaction vessel. H_2_ production was carried out *via* dispersing 20 mg of the photocatalyst into ultrapure water (50 mL) containing triethanolamine (5 mL) as a sacrificial electron donor in the reaction vessel. A certain amount of H_2_PtCl_6_ was dissolved in the reactant solution to deposit 1 wt% of Pt onto the catalyst. Before irradiation under visible light, the reaction system was evacuated several times to remove air completely. A flow of cooling water was used to maintain the temperature of the reaction device. The generated gases were analyzed by an on-line gas chromatograph (SHIMADZU, GC-8A) with a thermal conductivity detector (TCD) and argon was used as the carrier gas. The apparent quantum efficiency (AQE) was measured using a 300 W Xe lamp with a 420 nm band-pass filter. The AQE was calculated according to the following equation:^28^AQE (%) = (2 × *H*/*I*) × 100Here, *H* and *I* represent the number of the evolved H_2_ molecules and the number of incident photons, respectively. The number of incident photons was estimated by a calibrated Si photodiode, where all the incident photons were assumed to be absorbed by the photocatalyst. The stability of the materials was investigated by carrying out the photocatalytic reactions using the same procedure mentioned above with 40 mg catalyst for a total of 20 h with evacuation each 4 h.

## Results and discussion

**Fig. 1:**
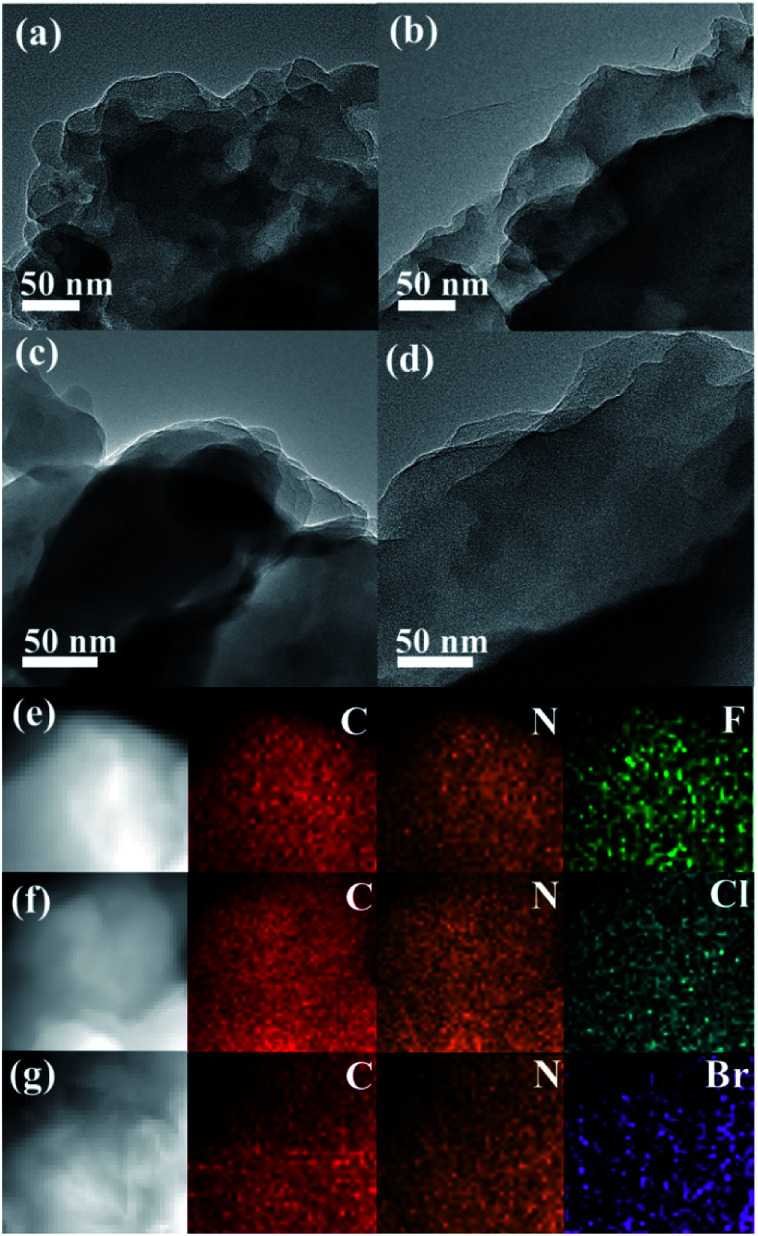
TEM images of (a) CTF-1, (b) CTFF, (c) CTFCl and (d) CTFBr. EDX elemental mappings of (e) CTFF, (f) CTFCl and (g) CTFBr.

As depicted in Fig. 1, the morphology analysis by TEM revealed that both CTF-1 and CTFX present a similar layered stacking structure, which can be further discerned in the SEM images (Fig. S1†). The EDX elemental mapping images of CTFX in Fig. 1e–g clearly demonstrated the homogeneous distribution of carbon, nitrogen and halogen in the CTFX samples. The type IV adsorption–desorption isotherms with a hysteresis loop for the CTF-1 and CTFX samples indicated the presence of a mesoporous structure (Fig. S2†). Notably, the similar specific surface areas and pore volumes of the CTF-1 and CTFX samples manifested that the effects of the surface area and pore structure on the photocatalytic activity can be neglected.

**Fig. 2:**
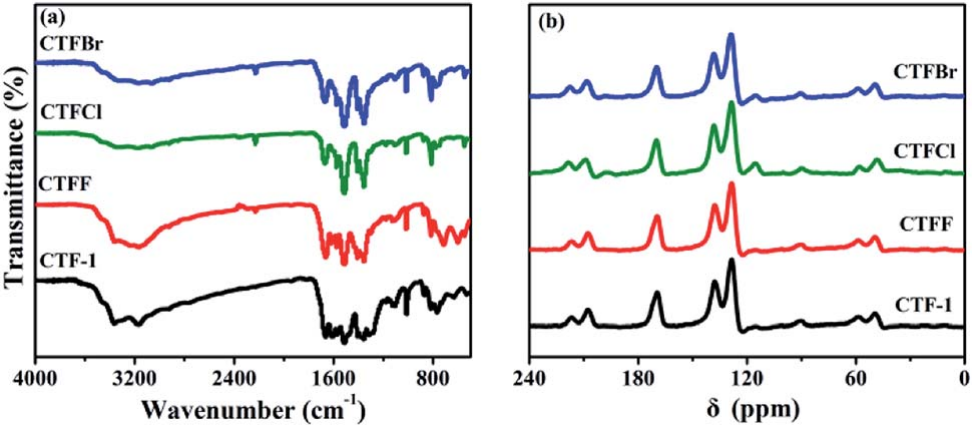
(a) FTIR spectra and (b) solid-state ^13^C NMR spectra of CTF-1 and CTFX samples.

The crystallinity of the as-obtained CTF-1 and CTFX samples was analyzed by obtaining powder XRD measurements (Fig. S3†). The diffraction peak intensity of the CTFX samples sharply decreased compared to that of CTF-1, suggesting that the layered CTF-1 exhibits a large extent of exfoliation after halogen doping.^29,30^ The maintained diffraction peak located at *ca.* 26.5°, which was assigned to the stacking of the conjugated aromatic systems, implied that the graphitic layers were delaminated without much lattice expansion or basal plane damage during the thermal treatment.^23^ Pure CTF-1 and CTFX samples displayed similar characteristic FT-IR vibration bands, as shown in Fig. 2a. The peaks at around 1508 cm^−1^ and 1357 cm^−1^ were assigned to the stretching vibration modes of the aromatic CN heterocycles.^31^ In comparison with the observation for CTF-1, an additional peak at 2228 cm^−1^ attributed to the cyanogroup (C≡N) was observed for CTFX samples.^8^ This might be induced by the partial thermal decomposition of CTF-1. Similar peaks were observed in the solid-state ^13^C NMR spectra of both the CTF-1 and CTFX samples (Fig. 2b). The peak at *ca.* 170 ppm is related to the sp^2^ carbon in the triazine ring, while the peaks at 138 and 128 ppm are attributed to the carbon in benzene rings.^31,32^ The FT-IR and NMR results demonstrated the good conservation of the conjugated backbone structure after halogen doping.

**Fig. 3:**
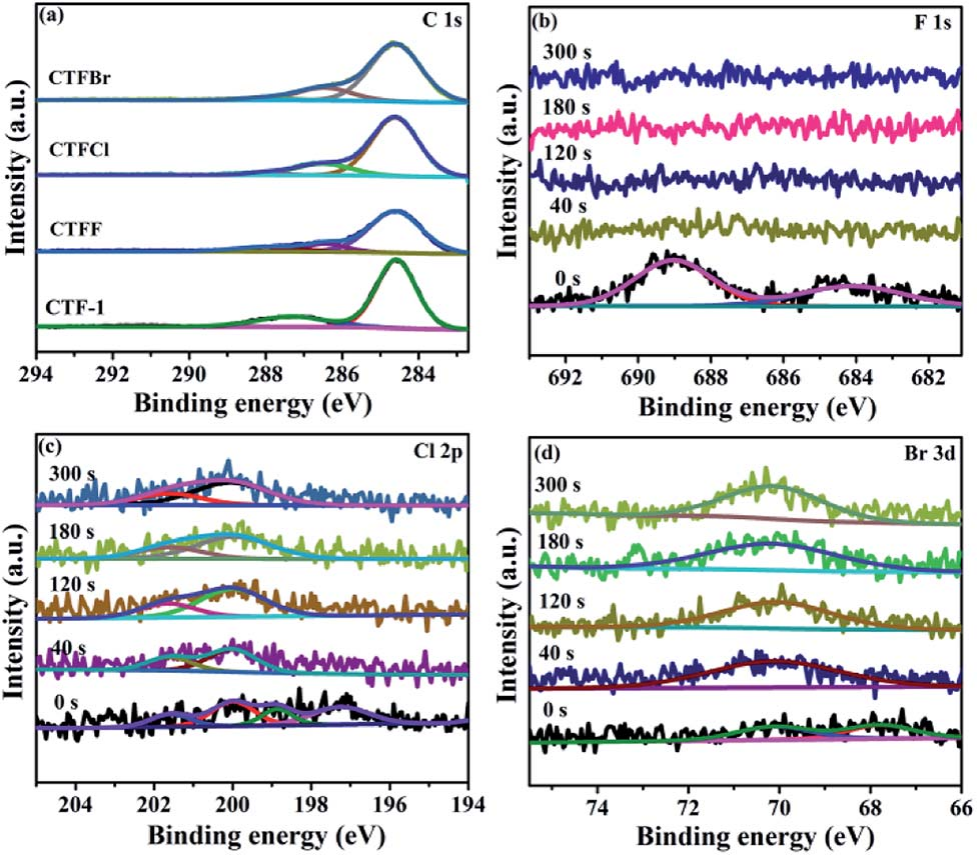
(a) C 1s XPS spectra of CTF-1 and CTFX samples. (b) F 1s XPS spectra of CTFF, (c) Cl 2p XPS spectra of CTFCl and (d) Br 3d XPS spectra of CTFBr after Ar^+^ sputtering from 0 to 300 s.

X-ray photoelectron spectroscopy (XPS) provided fundamental information regarding the chemical state and the doping level of the halogen elements in the CTFX samples. It was determined that the atomic concentration of halogens was 0.71 at% for CTFF, 0.36 at% for CTFCl and 0.19 at% for CTFBr. In the C 1s XPS spectra, the peaks at 284.6 eV, 286.6 eV and 288.0 eV were assigned to the C–C, C≡N and N–C=N bonds, respectively (Fig. 3a).^33^ Notably, the peak at 286.6 eV could also be attributed to the C–Cl bond and C–Br bond for the CTFCl and CTFBr samples, respectively.^34–36^ However, the corresponding C–F bond (288.7 eV) in the C 1s XPS spectrum of CTFF was not observed.^37^ The pristine CTF-1 sample exhibited a single peak at *ca.* 399.2 eV in the N 1s XPS spectrum, corresponding to the sp^2^-hybridized aromatic N bonded to C atoms (Fig. S4†).^38^ However, the N 1s peak of the CTFX samples shifted towards a lower binding energy compared with that of CTF-1, which was probably due to the change in the chemical environment after halogen doping. In the F 1s spectra of the CTFF sample, the weak peak at 684.0 eV was assigned to F^−^, while the strong peak at 689.0 eV was ascribed to F atoms in a semi-ionic C–F bond (Fig. 3b).^39^ It can be seen that these two peaks disappeared after Ar^+^ sputtering, suggesting that the F atoms were only distributed throughout the subsurface region with very limited depth. The XPS spectra of the Cl 2p peaks for the CTFCl sample can be split into two pairs, where the doublet located at 200.0 eV (2p_3/2_) and 201.6 eV (2p_1/2_) can be assigned to the covalent C–Cl bond; moreover, the lower binding energies at 197.1 eV (2p_3/2_) and 198.7 eV (2p_1/2_) corresponded to the ionic state of Cl (Fig. 3c).^35,40^ As shown in Fig. 3d, the XPS spectra of Br 3d can be fitted into two peaks. The peak located at 70.1 eV was assigned to the C–Br bond, while the peak at 67.8 eV was attributed to Br^−^.^22,32^ It is worth noting that the peaks corresponding to the C–Cl and C–Br bonds were well maintained after a long Ar^+^ sputtering time, demonstrating the homogeneous distribution of Cl and Br in CTFs, respectively.^41^ XPS analysis revealed that halogen atoms exhibited two chemical states, namely, halogens bonded with carbon in the frameworks of CTF-1 and the ionic halogens adsorbed on the surface of CTF-1.^21^

**Fig. 4:**
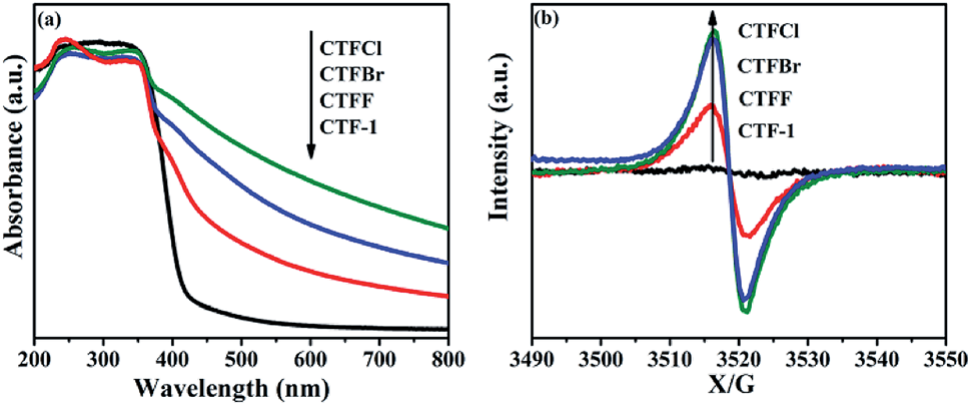
(a) UV-vis DRS spectra and (b) room-temperature EPR spectra in dark condition of CTF-1 and CTFX samples.

**Scheme 1:**
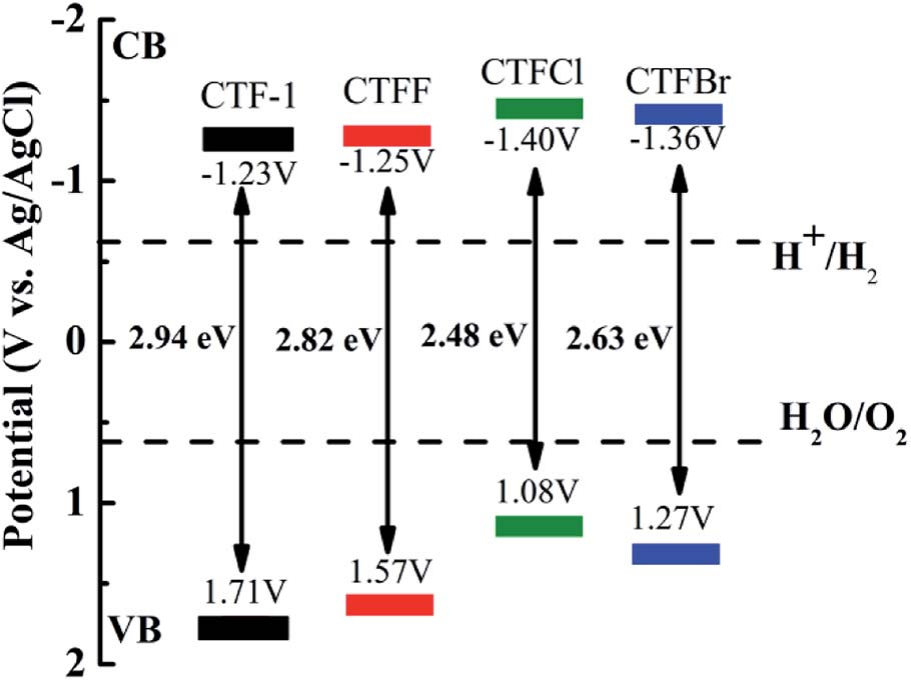
Electronic band structure *versus* the saturated Ag/AgCl reference electrode at pH = 7 for CTF-1 and the CTFX samples.

The optical properties of CTF-1 and CTFX were studied by UV-vis DRS (Fig. 4a). In comparison with the result for pristine CTF-1, additional absorption extending to longer wavelengths was observed for all CTFX samples. Furthermore, a weak red shift of the light absorption edge was observed for the halogen-doped CTF-1, suggesting a narrowed band gap of the CTFX samples. Based on the Kubelka–Munk function, the corresponding band gap energies of CTF-1, CTFF, CTFCl and CTFBr were calculated to be 2.94, 2.82, 2.48 and 2.63 eV. The narrowed band gaps of the CTFX samples were favourable for capturing more photons, which potentially contributed to the improved photocatalytic activities.^42^ The conduction band (CB) potentials of CTF-1 and CTFX were determined by their corresponding Mott–Schottky plots (Fig. S5†). The flat-band potentials obtained by the extrapolation of the Mott–Schottky plots were approximately −1.23 V for CTF-1, −1.25 V for CTFF, −1.40 V for CTFCl and −1.36 V for CTFBr *vs.* the saturated Ag/AgCl reference electrode at pH = 7. Based on the above UV-vis DRS spectra and the results of the Mott–Schottky plots, the electronic band structure diagrams of the CTF-1 and CTFX samples were proposed, as illustrated in Scheme 1. The band structures of the CTFX samples were thermodynamically suitable for photocatalytic hydrogen evolution under visible-light irradiation.

The presence of halogen functional groups on the aromatic ring has a significant impact on the electrical properties of CTF-1, which was determined by the room-temperature EPR spectra together with CTF-1 as a reference. In Fig. 4b, a single Lorentzian line centred at a *g*-value of 2.0034 is seen in the dark for all samples, which originates from the unpaired electrons on the aromatic rings.^43^ The EPR intensities were greatly enhanced after doping with halogens, and the intensities followed the order CTFCl > CTFBr > CTFF > CTF-1. The enhanced EPR intensities of the CTFX samples demonstrated that the modification with halogen atoms can effectively accelerate the electron mobility in the π-conjugated system of CTF-1, which might be due to the delocalization of the valence electrons of the halogen atoms on the conjugation system of CTF-1. The EPR experiments on the CTFX samples were also carried out under visible-light irradiation. Noticeably, enhanced intensities were detected compared with those under dark conditions (Fig. S6†), suggesting the efficient production of photochemical radical pairs in the CTFX samples.^44^

**Fig. 5:**
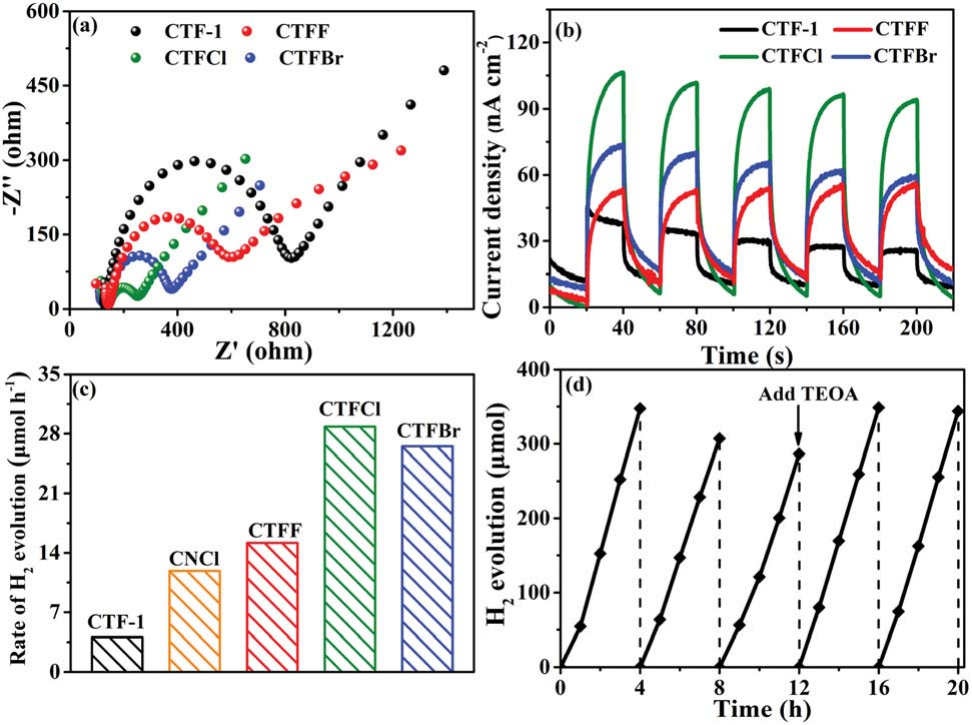
(a) Electrochemical impedance spectroscopy plots of CTF-1 and CTFX samples. (b) Photocurrent responses under visible-light irradiation of CTF-1 and CTFX samples. (c) H_2_ evolution rates of CTF-1, CNCl and CTFX samples. (d) The recycling performance of CTFCl for hydrogen evolution in five repeats.

To study the electron-transfer efficiency of halogen-doped CTF-1, electrochemical impedance spectroscopy (EIS) plots were measured in a 0.2 M Na_2_SO_4_ aqueous solution in the dark. As shown in Fig. 5a, markedly decreased Nyquist plot diameters can be observed for the CTFX samples relative to that for CTF-1, illustrating that halogen doping can effectively promote interfacial charge migration. The transient photocurrent responses of CTF-1 and CTFX with several on–off cycles are displayed in Fig. 5b. The CTFX samples showed higher photocurrent density than CTF-1 under visible-light illumination, which was in agreement with the EIS results. This illustrated the greatly enhanced charge separation and transfer efficiency of the CTFX samples, which more electrons and holes could participate in the subsequent reaction.^21^

The influence of halogen modification on the photocatalytic activity of CTF-1 was evaluated by photocatalytic hydrogen evolution under visible-light illumination (*λ* ≥ 420 nm). The photocatalytic reactions were carried out using 20 mg of the CTFX photocatalysts. As summarized in Fig. 5c, the rates of H_2_ evolution are greatly enhanced after halogen doping compared to that of un-doped CTF-1 and follow the order CTFCl > CTFBr > CTFF > CTF-1. The photoactivity obtained by CTFCl was about 7.1 times higher than that of unmodified CTF-1 and it was also superior to that of Cl-doped g-C_3_N_4_ (CNCl). The apparent quantum efficiency (AQE) of the H_2_-evolving half-reaction for optimal CTFCl was calculated to be 10.31%, demonstrating the superiority of the halogen-doped CTF-1 photocatalysts. Notably, the H_2_ evolution rates for the physical mixtures of CTF-1 and ammonium halide (CTFX-m, X = F, Cl and Br) were similar to that of pure CTF-1. This implied that the effects of the halogen ions adsorbed on the surface of CTF-1 on the photocatalytic performance can be neglected and the enhanced catalytic performances of the CTFX samples were derived from the introduction of halogen atoms into the framework of CTF-1 (Fig. S7†). On the basis of the above experimental results, a photocatalytic H_2_-production mechanism for CTFX can be proposed. The primary factors in a photocatalytic reaction are as follows: light absorption, photoexcitation to produce electron–hole pairs, electron–hole pair separation/migration and redox reactions.^45^ The UV-vis DRS, EPR and photoelectrochemical results presented identical trends for the photocatalytic H_2_-production activity of CTFX, reflecting that halogen doping can significantly influence the generation, separation and transfer efficiency of electrons in CTF-1. Firstly, the optical properties of CTFX were optimized to harvest more visible light, thus promoting the generation of photogenerated electron–hole pairs. Secondly, the electronic structures were modified to accelerate the separation and transfer of photoinduced electron–hole pairs. Moreover, the electron–hole pairs of CTFX could transfer to the surface of the photocatalysts more rapidly along the interlayer direction due to the ultrathin thickness of CTFX. This was verified by the exfoliation of CTFX, confirming the PXRD results. Finally, the textural structures were tailored, resulting in a more negative conduction band (CB) level of CTFX, which was proven by the Mott–Schottky plots. The more negative CB levels indicated the formation of photoelectrons with more powerful reducing ability, which was favourable for the improvement in the photocatalytic activity. It is worth noting that the surface areas and pore volumes of the CTFX samples were similar to those of pristine CTF-1, demonstrating that the effects of surface area and pore structure on the photocatalytic activity can be neglected. Therefore, the introduction of halogens into the frameworks of CTF-1 simultaneously modulated the crystal, optical, electronic and textural structures to optimize the four consecutive steps in the photocatalytic H_2_-production process, leading to strong synergetic enhancement in the photocatalytic activity.

The difference in photocatalytic H_2_ evolutions among different halogen dopants (F, Cl and Br) can be explained by two aspects: the electronegativity and the atomic size of the halogen atoms. In detail, the valence electrons with much lower electronegativity were more delocalized/mobile and could interact with the π-electron system of CTF-1 (F: 3.98; Cl: 3.16; Br: 2.96).^46^ Such an extended π-conjugated system of CTF-1 was beneficial for the mobility of the photo-generated carriers. This was proven by the EPR characterization and photoelectrochemical results, thus leading to different enhancements in the photocatalytic activities for the CTFX samples. However, due to the very large atomic sizes, it was more difficult to form thermodynamically and geometrically stable structures (C: 0.65 Å; N: 0.54 Å; F: 0.41 Å; Cl: 0.78 Å; Br: 1.03 Å). This somewhat counteracted the beneficial effects of the doping treatment. The XPS results showed that CTFBr exhibited the least concentration of the halogen element among the CTFX samples, which was in agreement with the deduction. Therefore, Cl atoms with appropriate electronegativity and atomic radius, which are close to those of the C and N atoms, respectively, would be the best halogen dopant to optimize the photocatalytic performance of CTF-1.

The stability of a photocatalyst is also a concern for the applications of polymer-based semiconductors. As a representative example of the CTFX series of samples, a cyclic H_2_ evolution test of optimal CTFCl (40 mg) was carried out under consecutive visible-light irradiations (*λ* ≥ 420 nm). Indeed, the active stability of CTFCl was rather excellent and no obvious deactivation was detected after 20 h of continuous irradiation (Fig. 5d). The used CTFCl sample was further subjected to characterizations by FT-IR and XPS for the investigation of chemical stability. The results shown in Fig. S8† revealed that there is no obvious change in the chemical structure before and after the reaction, demonstrating the excellent activity and chemical stability of the CTFX samples.

## Conclusions

Halogen-doped covalent triazine-based frameworks were successfully prepared using a facile thermal annealing method. The photocatalytic H_2_ evolution rates were greatly enhanced after doping with halogens (F, Cl and Br); in particular, CTFCl displayed optimal photocatalytic activity, which was about 7.1 times and 2.4 times higher than those of unmodified CTF-1 and Cl-doped g-C_3_N_4_, respectively. It was demonstrated that the narrowed band gaps, more negative CB positions and highly facilitated separation/transfer of charge carriers were responsible for the dramatically enhanced photocatalytic activity. This work paves a simple way for the further development of CTFs, and it also provides meaningful guidance for the design of non-metal-doped CTF photocatalysts for future practical applications.

## Conflicts of interest

There are no conflicts to declare.

## Supplementary Material

NA-001-C9NA00089E-s001

## References

[cit1] Zhang G. G., Lan Z. A., Wang X. C. (2016). Angew. Chem., Int. Ed..

[cit2] Diercks C. S., Yaghi O. M. (2017). Science.

[cit3] Sun S. D., Liang S. H. (2017). Nanoscale.

[cit4] Huang W., Byun J., Rorich I., Ramanan C., Blom P. W. M., Lu H., Wang D., da Silva L. C., Li R., Wang L., Landfester K., Zhang K. A. I. (2018). Angew. Chem., Int. Ed..

[cit5] Algara-Siller G., Severin N., Chong S. Y., Bjorkman T., Palgrave R. G., Laybourn A., Antonietti M., Khimyak Y. Z., Krasheninnikov A. V., Rabe J. P., Kaiser U., Cooper A. I., Thomas A., Bojdys M. J. (2014). Angew. Chem..

[cit6] Zhang L. S., Ding N., Wu J. H., Iwasaki K., Lin L. H., Yamaguchi Y. C., Shibayama Y. k., Shi J. J., Wu H. J., Luo Y. H., Nakata K., Li D. M., Wang X. C., Fujishima A., Meng Q. B. (2018). Catal. Sci. Technol..

[cit7] Cao S. W., Yu J. G. (2014). J. Phys. Chem. Lett..

[cit8] Kuhn P., Antonietti M., Thomas A. (2008). Angew. Chem., Int. Ed..

[cit9] Ren S., Bojdys M. J., Dawson R., Laybourn A., Khimyak Y. Z., Adams D. J., Cooper A. I. (2012). Adv. Mater..

[cit10] Jiang Q. Q., Sun L., Bi J. H., Liang S. J., Li L. Y., Yu Y., Wu L. (2018). ChemSusChem.

[cit11] Zhang G. G., Lin L. H., Li G. S., Zhang Y. F., Savateev A., Zafeiratos S., Wang X. C., Antonietti M. (2018). Angew. Chem., Int. Ed..

[cit12] Li L. Y., Li X. F., Cheng Z., Bi J. H., Liang S. J., Zhang Z. Z., Yu Y., Wu L. (2018). Dalton Trans..

[cit13] Jiang X., Wang P., Zhao J. J. (2015). J. Mater. Chem. A.

[cit14] Bi J. H., Fang W., Li L. Y., Wang J. Y., Liang S. J., He Y. H., Liu M. H., Wu L. (2015). Macromol. Rapid Commun..

[cit15] Ma X. G., Lv Y. H., Xu J., Liu Y. F., Zhang R. Q., Zhu Y. F. (2012). J. Phys. Chem. C.

[cit16] Li L. Y., Fang W., Zhang P., Bi J. H., He Y. H., Wang J. Y., Su W. Y. (2016). J. Mater. Chem. A.

[cit17] Bouša D., Pumera M., Sedmidubský D., Šturala J., Luxa J., Mazáneka V., Sofer Z. (2016). Nanoscale.

[cit18] Cao S. W., Low J. X., Yu J. G., Jaroniec M. (2015). Adv. Mater..

[cit19] Wei F. Y., Liu Y., Zhao H., Ren X. N., Liu J., Hasan T., Chen L. H., Li Y., Su B. L. (2018). Nanoscale.

[cit20] Wang H., Zhang X. D., Xie J. F., Zhang J. J., Ma P., Pan B., Xie Y. (2015). Nanoscale.

[cit21] Xiong T., Wong H., Zhou Y., Sun Y. J., Cen W. L., Huang H. W., Zhang Y. X., Dong F. (2018). Nanoscale.

[cit22] Lan Z. A., Zhang G. G., Wang X. C. (2016). Appl. Catal., B.

[cit23] Jeon I. Y., Choi H. J., Choi M., Seo J. M., Jung S. M., Kim M. J., Zhang S., Zhang L. P., Xia Z. H., Dai L. M., Park N. J., Baek J. B. (2013). Sci. Rep..

[cit24] Zhou J., Lin P., Ma J. J., Shan X. Y., Feng H., Chen C. C., Chen J. R., Qian Z. S. (2013). RSC Adv..

[cit25] Li J. D., Zhang X. L., Raziq F., Wang J. S., Liu C., Liu Y. D., Sun J. W., Yan R., Qu B. H., Qin C. L., Jing L. Q. (2017). Appl. Catal., B.

[cit26] Zhu B. C., Zhang J. F., Jiang C. J., Cheng B., Yu J. G. (2017). Appl. Catal., B.

[cit27] Lin Q. Y., Li L., Liang S. J., Liu M. H., Bi J. H., Wu L. (2015). Appl. Catal., B.

[cit28] Maeda K., Teramura K., Lu D. L., Takata T., Saito N., Inoue Y., Domen K. (2006). Nature.

[cit29] Han Q., Hu C. G., Zhao F., Zhang Z. P., Chen N., Qu L. T. (2015). J. Mater. Chem. A.

[cit30] Yang S. B., Gong Y. J., Zhang J. S., Zhan L., Ma L. L., Fang Z. Y., Vajtai R., Wang X. C., Ajayan P. M. (2013). Adv. Mater..

[cit31] Bhunia A., Esquivel D., Dey S., Fernández-Terán R., Goto Y., Inagaki S., Van Der Voort P., Janiak C. (2016). J. Mater. Chem. A.

[cit32] Kuecken S., Acharjya A., Zhi L. J., Schwarze M., Schomacker R., Thomas A. (2017). Chem. Commun..

[cit33] Zhang G. G., Zhang J. S., Zhang M. W., Wang X. C. (2012). J. Mater. Chem..

[cit34] Li B., Zhou L., Wu D., Peng H. L., Yan K., Zhou Y., Liu Z. F. (2011). ACS Nano.

[cit35] Wu Y., Lin X. Y., Shen X., Sun X. Y., Liu X., Wang Z. Y., Kim J. K. (2015). Carbon.

[cit36] Huang G. B., Song P. G., Liu L., Han D. M., Ge C. H., Li R. R., Guo Q. P. (2016). Carbon.

[cit37] Liu Y., Feng Q., Xu Q. H., Li M., Tang N. J., Du Y. W. (2013). Carbon.

[cit38] Luo Z. Q., Lim S. H., Tian Z. Q., Shang J. Z., Lai L. F., MacDonald B., Fu C., Shen Z. X., Yu T., Lin J. Y. (2011). J. Mater. Chem..

[cit39] Qiao X. C., Liao S. J., Wang G. H., Zheng R. P., Song H. Y., Li X. H. (2016). Carbon.

[cit40] Li X. M., Lau S. P., Tang L. B., Ji R. B., Yang P. Z. (2013). J. Mater. Chem. C.

[cit41] Zheng J., Liu H. T., Wu B., Di C. A., Guo Y. L., Wu T., Yu G., Liu Y. Q., Zhu D. B. (2012). Sci. Rep..

[cit42] Fu J. W., Zhu B. C., Jiang C. J., Cheng B., You W., Yu J. G. (2017). Small.

[cit43] Zhang G. G., Zhang M. W., Ye X. X., Qiu X. Q., Lin S., Wang X. C. (2014). Adv. Mater..

[cit44] Fang J. W., Fan H. Q., Li M. M., Long C. B. (2015). J. Mater. Chem. A.

[cit45] Ran J. R., Ma T. Y., Gao G. P., Du X. W., Qiao S. Z. (2015). Energy Environ. Sci..

[cit46] Li Z., Tian B., Zhang W. Y., Zhang X. Q., Wu Y. Q., Lu G. X. (2017). Appl. Catal., B.

