# Supplementary material for: Constructing a novel family of halogen-doped covalent triazine-based frameworks as efficient metal-free photocatalysts for hydrogen production
Source: Nanoscale Adv. 2019 May 18;1(7):2674–80. doi: 10.1039/c9na00089e (PMC9418566; doi:10.1039/c9na00089e)
Supplement: NA-001-C9NA00089E-s001 [file NA-001-C9NA00089E-s001.pdf]

Electronic Supplementary Material (ESI) for Nanoscale Advances.
This journal is © The Royal Society of Chemistry 2019

Electronic Supplementary Material (ESI) for Nanoscale Advances.
This journal is © The Royal Society of Chemistry 2019

SUPPLEMENTARY INFORMATION

Constructing a novel family of halogen doped covalent triazine-based frameworks as efficient metal-free photocatalysts for hydrogen production

Zhi Cheng, [a] Kaiyun Zheng, [a] Guiyun Lin, [a] Shengqiong Fang, [a] Liuyi Li,[*,c]

Jinhong Bi,[*,a,b] Jinni Shen [c] and Ling Wu [b]

[a] Department of Environmental Science and Engineering, Fuzhou University, Fuzhou 350108, China

[b] State Key Laboratory of Photocatalysis on Energy and Environment, Fuzhou University, Fuzhou 350108, China

[c] Key Laboratory of Eco-materials Advanced Technology, Fuzhou University, Fuzhou 350108, China

*Corresponding author, E-mail: lyli@fzu.edu.cn; bijinhong@fzu.edu.cn

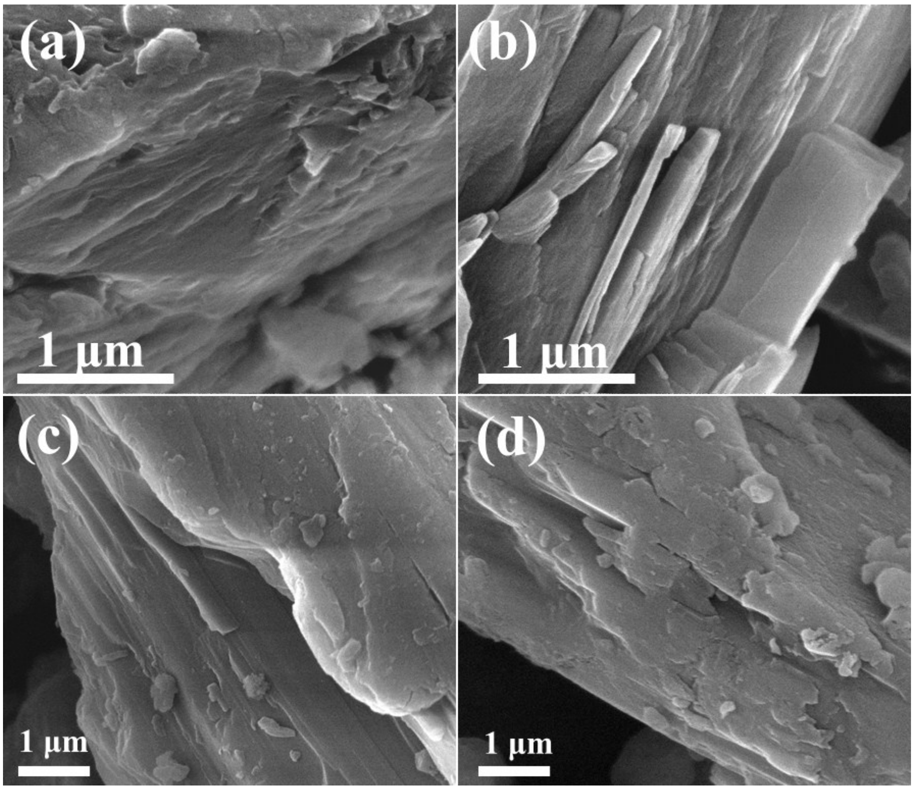

Fig. S1 SEM images of (a) CTF-1, (b) CTFF, (c) CTFCl and (d) CTFBr.

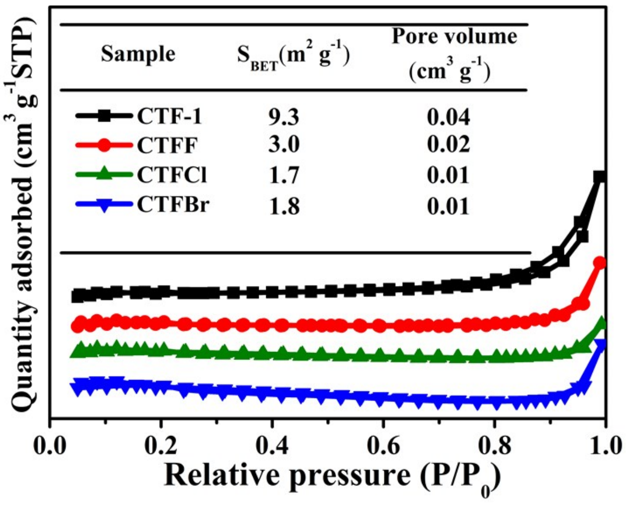

Fig. S2 N_2 adsorption-desorption isotherms of CTF-1 and CTFX samples and table of the specific surface areas and pore volumes (inset).

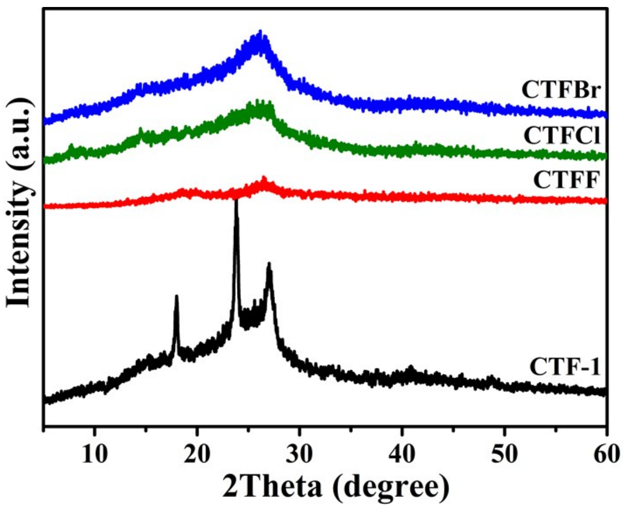

Fig. S3 PXRD patterns of CTF-1 and CTFX samples.

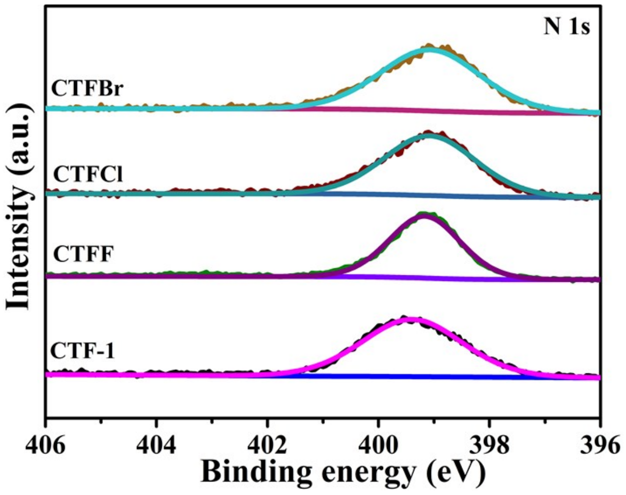

Fig. S4 N 1s XPS spectra of CTF-1 and CTFX samples.

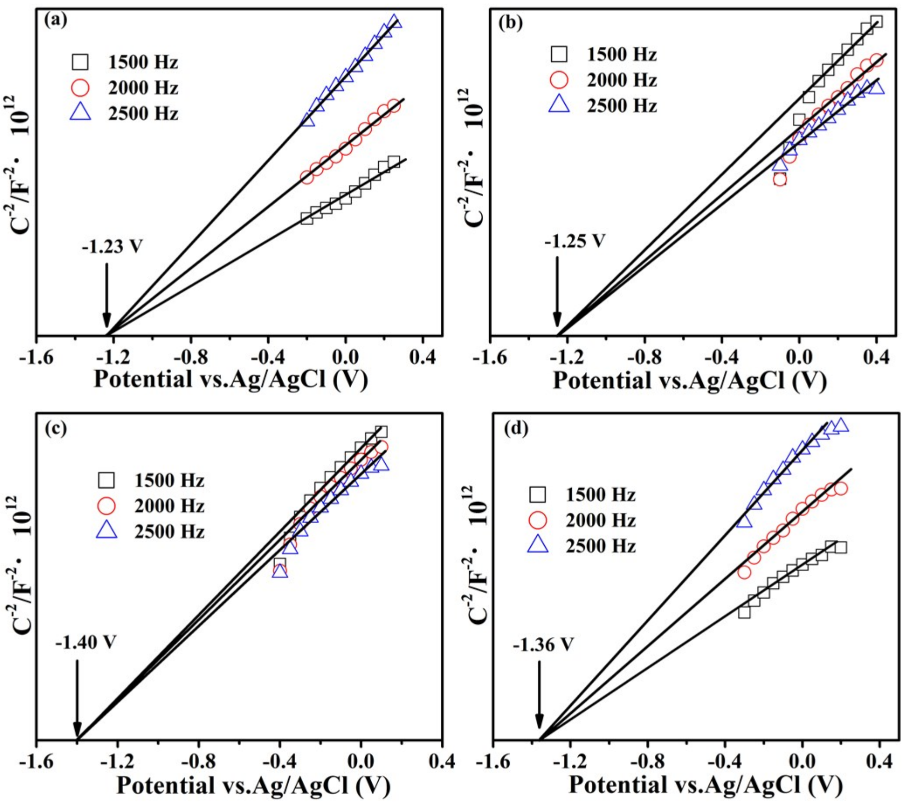

Fig. S5 Electrochemical Mott-Schottky plots of (a) CTF-1, (b) CTFF, (c) CTFCl and (d) CTFBr.

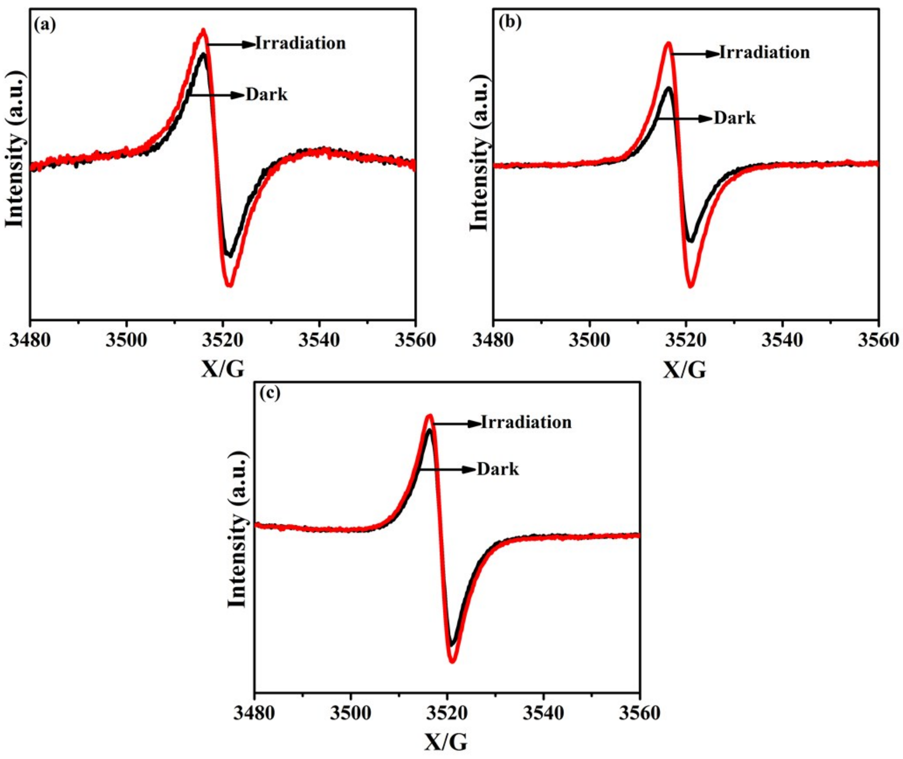

Fig. S6 EPR spectra of (a) CTFF, (b) CTFCl and (c) CTFBr in the dark and after visible light irradiation.

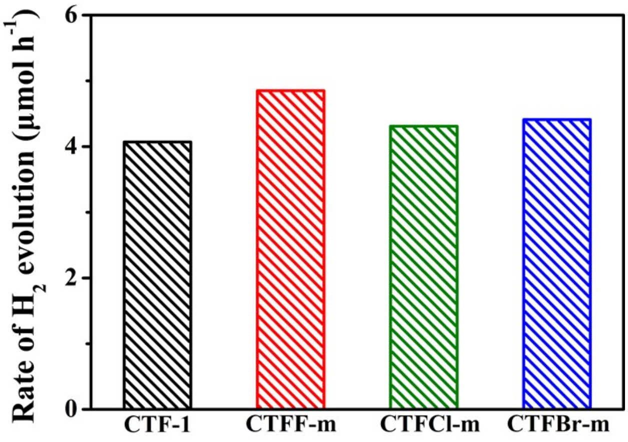

Fig. S7 H_2 evolution rates of CTF-1 and CTFX-m (X=F, Cl and Br).

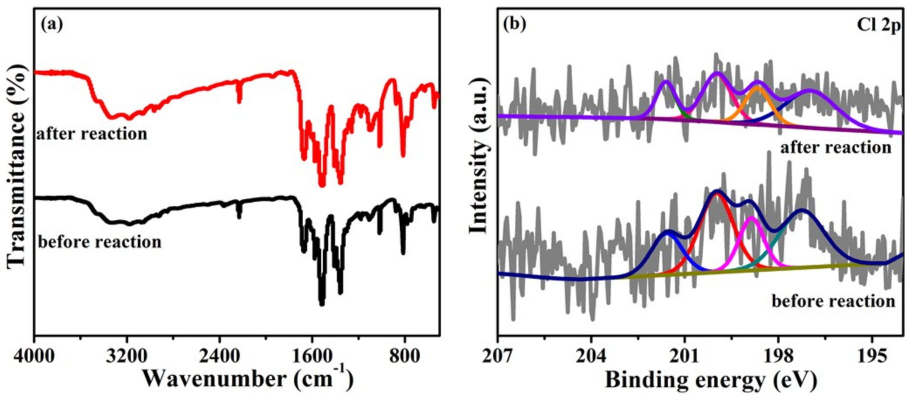

Fig. S8 (a) FT-IR spectra and (b) Cl 2p XPS spectra of CTFCl before and after catalytic reaction.
